# Synthetic RNAs Mimicking Structural Domains in the Foot-and-Mouth Disease Virus Genome Elicit a Broad Innate Immune Response in Porcine Cells Triggered by RIG-I and TLR Activation

**DOI:** 10.3390/v7072807

**Published:** 2015-07-17

**Authors:** Belén Borrego, Miguel Rodríguez-Pulido, Concepción Revilla, Belén Álvarez, Francisco Sobrino, Javier Domínguez, Margarita Sáiz

**Affiliations:** 1 Centro de Investigación en Sanidad Animal, CISA-INIA, Valdeolmos, Madrid 28130, Spain; E-Mail: borrego@inia.es; 2 Centro de Biología Molecular Severo Ochoa (CISC-UAM), Cantoblanco, Madrid 28049, Spain; E-Mails: mrrodriguez@cbm.csic.es (M.R.-P.); fsobrino@cbm.csic.es (F.S.); 3 Dpto. de Biotecnología, Instituto Nacional de Investigación y Tecnología Agraria y Alimentaria (INIA), Ctra de la Coruña Km 7.5, Madrid 28040, Spain; E-Mails: crevilla@inia.es (C.R.); avega@inia.es (B.Á.); juncal@inia.es (J.D.)

**Keywords:** antivirals, non-coding RNA, viral sensors, innate immunity, foot-and-mouth disease virus

## Abstract

The innate immune system is the first line of defense against viral infections. Exploiting innate responses for antiviral, therapeutic and vaccine adjuvation strategies is being extensively explored. We have previously described, the ability of small *in vitro* RNA transcripts, mimicking the sequence and structure of different domains in the non-coding regions of the foot-and-mouth disease virus (FMDV) genome (ncRNAs), to trigger a potent and rapid innate immune response. These synthetic non-infectious molecules have proved to have a broad-range antiviral activity and to enhance the immunogenicity of an FMD inactivated vaccine in mice. Here, we have studied the involvement of pattern-recognition receptors (PRRs) in the ncRNA-induced innate response and analyzed the antiviral and cytokine profiles elicited in swine cultured cells, as well as peripheral blood mononuclear cells (PBMCs).

## 1. Introduction

Activation of innate immune signaling pathways through pattern-recognition receptors (PRRs) in the host cell is a crucial step for defense against infection by pathogens. PRRs recognize pathogen-associated molecular patterns (PAMPs), chemical or structural features present in pathogens but not in host cells, which act as alert signals to the innate immune system of the host [1]. PAMPs present in viral genomes or generated during the course of a virus infection include single-stranded RNA (ssRNA) or double-stranded RNA (dsRNA). dsRNA is produced in infected cells as genomic fragments, replicative intermediate or by stem-loop structures and recognized by viral sensors [2]. Among the PRRs involved in sensing viral genomes, Toll-like receptors (TLRs), expressed on the surface and endosomal compartments of some cell types [3], and retinoic acid-inducible gene-I (RIG-I)-like receptors (RLRs), ubiquitous cytosolic RNA helicases [4,5,6], have a remarkable role in detection of viral RNA. The RLRs RIG-I and melanoma differentiation-associated gene 5 (MDA5) play critical roles in triggering immune defenses against RNA virus infection. When the inactive forms of RIG-I or MDA5 bind viral RNA, the helicases undergo a conformational change, multimerization and then, interact with the adaptor molecule MAVS through their CARD domains, triggering a signaling cascade to activate the expression of type-I IFN and proinflammatory cytokines through IFN-regulatory factors 3 and 7 (IRF 3/7) and NF-κB leading to the establishment of an antiviral state within infected cells and in neighboring non-infected cells. RIG-I and MDA5 recognize distinct RNA species that have reached the cytoplasm by infection or by means of transfection. How RLRs discriminate such RNAs is still a topic of intense study and some specific primary, secondary and tertiary structures have been identified [5]. It is widely accepted that RIG-I ligands include ssRNA bearing a 5′-triphosphate and a blunt-ended region at the 5′end, as well as short dsRNA, while MDA5 binding is associated to long dsRNA [5,7].

Of the TLRs characterized to date, several have been linked to antiviral immunity. Among these, TLR3, TLR7, TLR8, and TLR9 detect distinct forms of viral nucleic acids and are critical in the recognition of viral genetic materials in endolysosomal compartments and initiation of antiviral responses [8]. TLR3 recognizes dsRNA, TLR7 and TLR8 detect ssRNA, while TLR9 recognizes unmethylated CpG DNA. Initially localized to the endoplasmic reticulum after their synthesis, these TLRs depend on the membrane protein UNC93B1 for transport to endolysosomal compartments where they are processed to become functional receptors. All TLRs signal through MyD88 (myeloid differentiation primary response 88) except TLR3, which only signals through TRIF (TIR-domain containing adaptor inducing interferon (IFN)-β). Like TLR3 and TLR9, recognition of viral ligands in the endosome by TLR7/TLR8 is dependent on acidification of the endosomal vesicles [8,9].

New vaccine adjuvants are designed to improve the recruitment and activation of dendritic cells, then enabling transition from the innate to adaptive immune system for priming of B- and T-cell responses. Endogenous or therapeutically induced early type-I IFN responses may confer protection until adaptive immunity is activated to an extent that the pathogen can be eliminated. In that context, PRRs come into sight as targets of new vaccine adjuvants besides their role as sentinels in innate immunity [10,11]. In previous work, we have described the ability of small RNA transcripts corresponding to different domains in the non-coding regions (NCRs) of the foot-and-mouth disease virus (FMDV) genome (ncRNAs) to induce a robust innate immune response and antiviral activity against a range of viral pathogens in cultured cells and mice [12,13,14,15]. These regions include the 5′-terminal S fragment (S), the internal ribosome entry site (IRES) and the 3′-terminal region and attached poly (A) tail (3′NCR). All of them are known or predicted to play relevant functions in the viral cycle and based on *in silico* analysis, as well as molecular probing, they are predicted to display a strong and defined secondary structure with stable hairpin and stem-loop motifs [16,17,18,19]. The potential application of these synthetic non-infectious RNA molecules as new immunomodulatory or adjuvant compounds for FMD vaccine improvement has been proposed according to promising results in a mice model [20]. Here, we have investigated the innate immune response induced by the FMDV ncRNAs in swine peripheral blood mononuclear cells (PBMCs) and the signaling pathways involved. We show that the FMDV ncRNAs are able to trigger a broad innate immune response in swine PBMCs and identified the RLR and TLR signaling pathways as sensors accounting for the induced type-I IFN and cytokine response.

## 2. Materials and Methods

### 2.1. Cells and Plasmids

Human embryonic kidney HEK 293 cells (kindly donated by E. Martínez-Salas, Centro de Biología Molecular Severo Ochoa, Madrid, Spain) and swine kidney SK6 cells (obtained from Centro de Investigación en Sanidad Animal, CISA-INIA, Valdeolmos, Spain) were grown in Dulbecco’s modified Eagle’s medium supplemented with 5%–10% fetal calf serum (FCS), penicillin-streptomycin (pen-strep), and l-glutamine.

The primary swine cells used were total PBMCs and PBMCs enriched in plasmacytoid dendritic cells (pDCs). Blood was collected from Landrace x Large White pigs in 5 mM EDTA, diluted 1:1 in PBS and then used to obtain PBMCs by density-gradient centrifugation with Histopaque 1077 (Sigma). In some cases, PBMCs were isolated on Percoll discontinuous gradients after blood sedimentation through dextran, as previously described [21]. Trypan blue was used to assess viability. PBMCs were resuspended in RPMI 1640 medium, containing 10% FCS, 20 mM HEPES, 2 mM l-glutamine, 50 μM 2-mercaptoethanol, pen-strep (100 U/mL–0.1 mg/mL) and seeded in 48- or 24-well plates at a density of 2.5–3 million cells per mL. pDCs were enriched by depleting PBMCs of monocytes and T lymphocytes using monoclonal antibodies to porcine CD11R3 (2F4/11, IgG1) and porcine CD3 (BB23-8E, IgG2b) bound to magnetic beads and the magnetic-activated cell-sorting (MACS) system (Miltenyi Biotec).

Monoclonal antibody to porcine CD3 was kindly provided by M. Pescovitz (Indiana University, USA). Monoclonal antibody to CD11R3 was developed in our laboratory. The negative selection procedure used yields around a 10-fold enrichment in pDCs corresponding to a 3%–6% of pDCs in the PBMC population.

Expression plasmids for flag-tagged full-length MDA5, RIG-I, and constitutively active RIG-I C-terminal deletion mutant (2CARD) generated by cloning the corresponding human sequences into pCAGGS-Flag [22,23], as well as reporter plasmid expressing firefly luciferase under the human IFN-β promoter [24] were all kindly provided by A. García-Sastre (Mount Sinai School of Medicine, New York, NY, USA).

### 2.2. RNA Synthesis

RNA transcripts corresponding to structural domains in the 5′ and 3′ NCRs of the FMDV genome (ncRNAs) were generated as described [12]. Briefly, RNA transcripts corresponding to the S fragment and 3′ NCR of the FMDV O1K genome were synthetized by *in vitro* transcription with T3 RNA polymerase (NEB) from previously described plasmids that were linearized with *Not*I [16]. RNA corresponding to the IRES of FMDV C-S8 was generated from a pGEM-derived clone [25], a gift of E. Martínez-Salas, after linearization with *Xho*I and *in vitro* transcription with T7 RNA polymerase (NEB). After transcription, RNAs were treated with RQ1 DNase (1 U/μg; Promega, Madison, WI, USA), extracted with phenol-chloroform, and precipitated with ethanol. All transcripts were resuspended in water and quantified by spectrometry. The RNA integrity and size were analyzed in denaturing 6% acrylamide, 7 M urea gel electrophoresis or agarose gels. Prior to transfection, RNAs were heated at 92 °C for 5 min, cooled down to room temperature for 10 min, and then chilled on ice.

### 2.3. Transfection of HEK 293 Cells and Luciferase Assay

Approximately 1 × 10^6^ HEK 293 cells were co-transfected with 50 ng of pIFNβ-Luc (firefly luciferase), 25 ng of renilla luciferase reporter plasmid (pRL-TK) constitutively expressing renilla luciferase (Promega) and different concentrations of RIG-I, MDA5, 2CARD or empty plasmid. After 24 h, cells were mock-treated or stimulated with increasing amounts of the different ncRNAs. Lipofectamine 2000 (Invitrogen, Waltham, MA, USA) was used for DNA and RNA transfection, according to manufacturer’s protocol. Cells were harvested 24 h later and dual-luciferase assay was performed using Dual-Luciferase Reporter Assay System (Promega). The relative firefly luciferase activity was normalized to Renilla luciferase activity and expressed as fold differences relative to mock-transfected cells.

### 2.4. Transfection of SK6 Cells and RT-PCR

Approximately 1 × 10^6^ SK6 cells were transfected with 20 μg/mL 3′NCR transcripts using Lipofectin (Invitrogen). Cells were lysed at different times after transfection and total RNA was extracted, quantified by spectrometry and DNase-treated with Turbo DNA-free kit (Ambion, Waltham, MA, USA). Then, aliquots of 300 ng RNA were used for RT-PCR analysis of Mx1, and 750 ng for IFN-β and GAPDH. The following primers were used for porcine Mx1 amplification: 5′-ACCAGGGTAGCTGTAGGCAA-3′ and 5′-ATCATGTAGCCCTTCTTCAG-3′; the 284-bp amplification product was analyzed on 2% agarose gels. For amplification of porcine IFN-β, primers used were: 5′-TTATCCACGAGATGCTCCAGCAG-3′ and 5′-GTGAAGAATGGTCATGTCTCCCC-3′; the 187-bp product was detected on 2.5% agarose gels. GAPDH amplification was performed using primers previously described [26].

### 2.5. PBMCs Transfection, RT-PCR and Cytokine Detection

Swine PBMCs (3 × 10^6^, unless otherwise specified) were transfected with the ncRNAs and RNA was extracted and treated with DNase, as described for SK6 cells. Then, aliquots of 20 ng of RNA were used for RT-PCR analysis of Mx1 (as described in 2.4) and cyclophilin for normalization using previously described primers [27]. In some cases, PBMCs were treated with bafilomycin A1 (250 nM, Sigma, St. Louis, WA, USA) 15–30 min prior to transfection with the ncRNAs or stimulated with polyinosine-polycytidylic acid (Poly I:C) (10 μg/μL, Sigma), LPS from E. coli 055:B5 (0.5 μg/μL, Sigma) or ODN 2216 (10 μg/μL, Invivogen) which are TLR-3, -4 and -9 specific ligands, respectively. Unless indicated otherwise, supernatants were collected 24 h after transfection or stimulation and aliquots were then kept at −80 °C. The levels of IFN-α, TNF-α, IL-10 and IL-12 in the supernatant of stimulated PBMCs were measured by ELISA. Commercial antibody pairs and reagents purchased from Invitrogen (TNF-α and IL-10) and R&D Systems (IL-12, Minneapolis, MN, USA) were used following the manufacturer’s instructions. For IFN-α, monoclonal antibodies K9 and F17 from PBL Interferon Source were used, as described [28]. In some experiments, type-I IFN activity was assessed by antiviral activity in the supernatants of transfected PBMCs by type-O FMDV infection inhibition assay on IBRS-2 cells, as described [12].

### 2.6. Ethical Statement

The protocols included in this study involving animal samples were approved by the Committee on the Ethics of Animal Experiments of INIA (permit number CEEA 2014-018). All animal care and handling strictly followed the current Spanish legislation (Ley 6/11 June 2013) and guidelines of the European Parliament and Council Directive (2010/63/EU).

### 2.7. Immunoblot Analysis

SK6 cells transfected with 3′NCR transcripts or HEK 293 cells transfected with flag-tagged plasmids expressing MDA5 or RIG-I were washed twice in ice-cold PBS and lysed at different times post-transfection (pt) in PBS containing 1% NP-40, 1 mM DTT, 1 mM phenylmethylsulfonyl fluoride and 1× Complete protease inhibitor cocktail (Roche, Basel, Switzerland). 20 μg of cell extracts was run on 10% SDS-PAGE gels, transferred onto nitrocellulose membrane and probed with the corresponding primary antibody. Blots were then incubated with the corresponding goat anti-mouse, goat anti-rabbit or rabbit anti-goat IgG (H + L) secondary antibody HRP conjugate (Thermo Scientific Pierce, Rockford, IL, USA). Protein bands were visualized using Western Lightning Plus-ECL detection reagents (Perkin Elmer Inc., Waltham, MA, USA) followed by exposure to X-ray film. Monoclonal anti-FLAG M2 (F1804) was purchased from Sigma. Mouse monoclonal anti-porcine Mx1 (AM39) was from Acris Antibodies. Goat polyclonal antibodies against human RIG-I (sc-48929) and MDA-5 (sc-48031) were purchased from Santa Cruz. Rabbit polyclonal antibody anti-βII tubulin has been previously described [29].

### 2.8. Statistical Analysis

The unpaired Student’s *t*-test was used to compare data among groups using GraphPad PRISM v.5.01 software (GraphPad Software Inc., La Jolla, CA, USA).

## 3. Results

### 3.1. The FMDV ncRNAs Activate the Human IFN-β Promoter in a RIG-I-Dependent Manner

**Figure 1:**
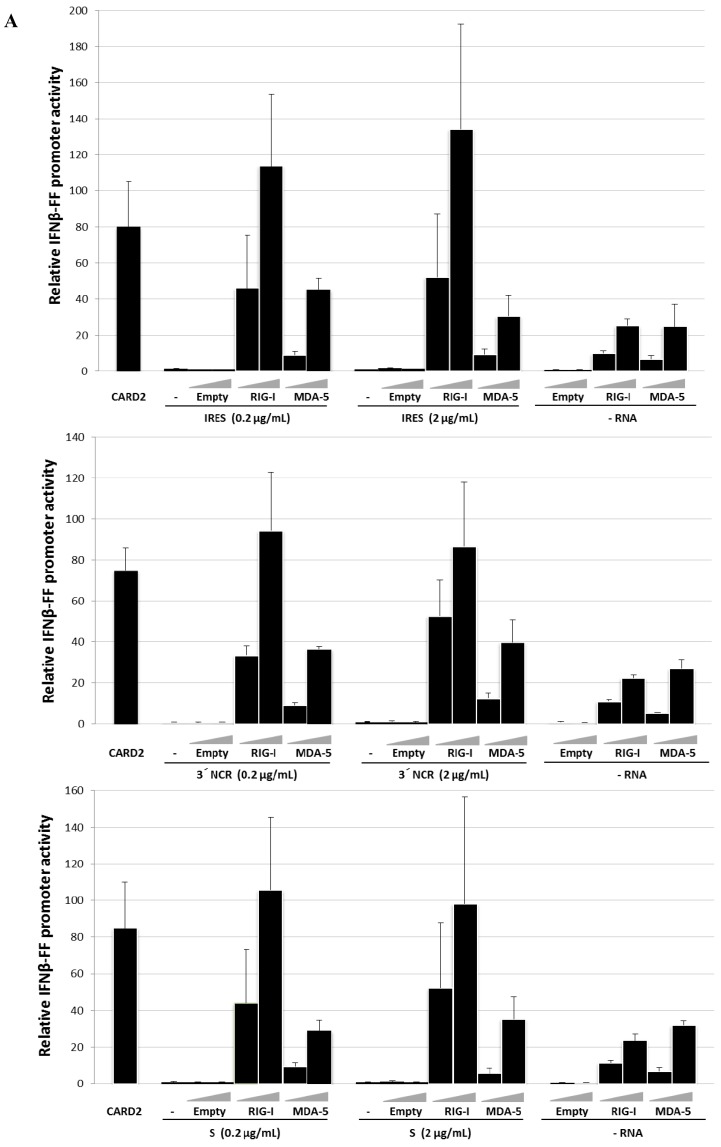

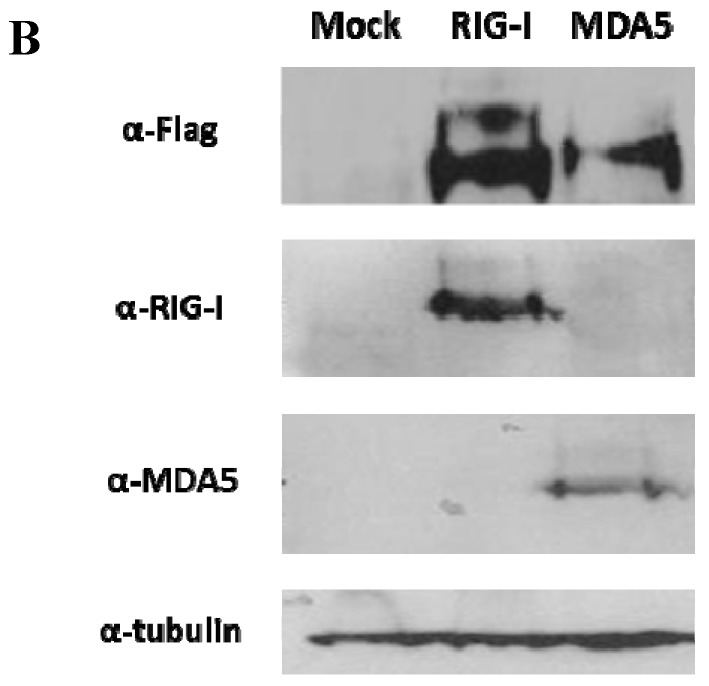
Induction of IFN-β promoter activity by the FMDV ncRNAs in HEK 293 cells expressing RLRs. (**A**) Cells (1 × 10^6^) were co-transfected with 50 ng of pIFNβ-FFLuc, 25 ng of pRL-TK, and increasing amounts of flag-RIG-I, flag-MDA5 or empty plasmid (1 and 10 ng). A constitutively active RIG-I, CARD2 (2 ng) served as positive control. After 24 h, cells were mock-treated or stimulated with 0.3 μg (0.2 μg/mL) or 3 μg (2 μg/mL) of the indicated ncRNA. Cells were harvested 24 h later and dual-luciferase assay was performed. The values represent the relative firefly luciferase activity normalized to Renilla luciferase activity and expressed as fold differences relative to mock-transfected cells. Data are representative of two independent experiments and error bars indicate mean +/− SD; (**B**) Expression of RLRs in transfected 293 cells. HEK293 cells were transfected with 100 ng of flag-RIG-I or flag- MDA5. Forty-eight hours later, cell lysates were prepared and analyzed by immunoblot with anti-flag, anti-RIG-I, anti-MDA5 or anti-β-tubulin antibodies.

To investigate the route of induction of the innate response triggered by the ncRNAs, we assayed the activation of the human IFN-β promoter in transfected HEK 293 cells expressing the members of the RLR family involved in viral RNA recognition. HEK 293 cells were transfected with expression plasmids containing the sequence of flag-tagged human RIG-I or MDA5 24 h before transfection with increasing amounts of the IRES, 3′NCR or S RNA transcripts. A constitutively active RIG-I C-terminal deletion mutant (2CARD) and the empty plasmid were used as positive and negative controls, respectively. The results of the IFN-β promoter activity relative to that of constitutive TK promoter after 24 h of RNA transfection are shown in Figure 1A. A specific increase in the IFN-β promoter activity induced by transfection with each ncRNA was detected when cells were previously transfected with RIG-I. Depending on the particular RNA and concentration used, this induction ranged between 3 to 5.3-fold relative to mock-transfected cells expressing the corresponding plasmid. The highest induction level was observed with the IRES RNA (5.3-fold), followed by the 3′NCR (4.8-fold) and the S RNA (4.6-fold) all of them at 2 μg /mL. However, expression of MDA5 did not yield a significant induction of the promoter activity after RNA transfection with values about 1.1 to 1.8 for all the ncRNAs. When cells were transfected with an empty vector, IFN-β transcription was not stimulated by the ncRNAs. The expression of tagged-RLRs in transfected 293 cells was confirmed by immunoblot 48 hpt using specific anti-Flag, -RIG-I and -MDA5 antibodies (Figure 1B). We conclude from these data that transfection with the S, IRES and 3′NCR transcripts triggers activation of the IFN-β promoter in human HEK 293 cells through a RIG-I dependent pathway.

### 3.2. Induction of Mx1 in Porcine Epithelial Cultured Cells Transfected with the 3′NCR RNA

**Figure 2:**
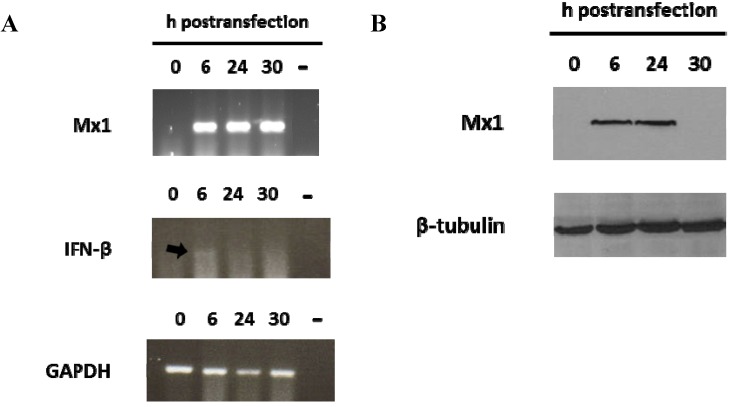
Mx1 induction in porcine cells transfected with the 3′NCR RNA. SK6 cells (1 × 10^6^) were transfected with 20 μg/mL 3′NCR transcripts. Cells were lysed at 0, 6, 24 or 30 h after transfection. (**A**) RT-PCR detection of IFN-β, Mx1 and GAPDH mRNAs in RNA extracted from SK6 lysates. Negative controls (water) were included in the RT-PCR assays; (**B**) Mx1 detection by immunoblot in transfected SK6 cells. Tubulin was used for normalization.

Since the FMDV ncRNAs were sensed by RIG-I in human embryonic kidney cells, the paracrine effect of the type-I IFN induced by ncRNAs transfection was analyzed in swine kidney SK6 cells. For that, detection of myxovirus resistance gene Mx1, an IFN-stimulated gene, was assayed at different times pt with the 3′NCR transcript, previously found to activate a robust IFN-α/β response in porcine cultured cells [12]. Mx proteins are key components of the antiviral state induced by IFNs in many species, some of them exerting antiviral activity against a wide range of RNA viruses [30]. Induction of the swine Mx1 mRNA could be clearly detected by RT-PCR at 6 hpt, coincident with a peak of IFN-β mRNA induction (detected as a faint band) (Figure 2A). The induction was maintained at least until 30 hpt. The Mx1 protein could also be detected by immunoblot in transfected cells from 6 to 24 hpt (Figure 2B). These results indicate that ncRNA transfection triggers a rapid and effective innate response with a potential antiviral effect.

### 3.3. The FMDV ncRNAs Induce Antiviral and Cytokine Responses in Porcine PBMCs

**Figure 3:**
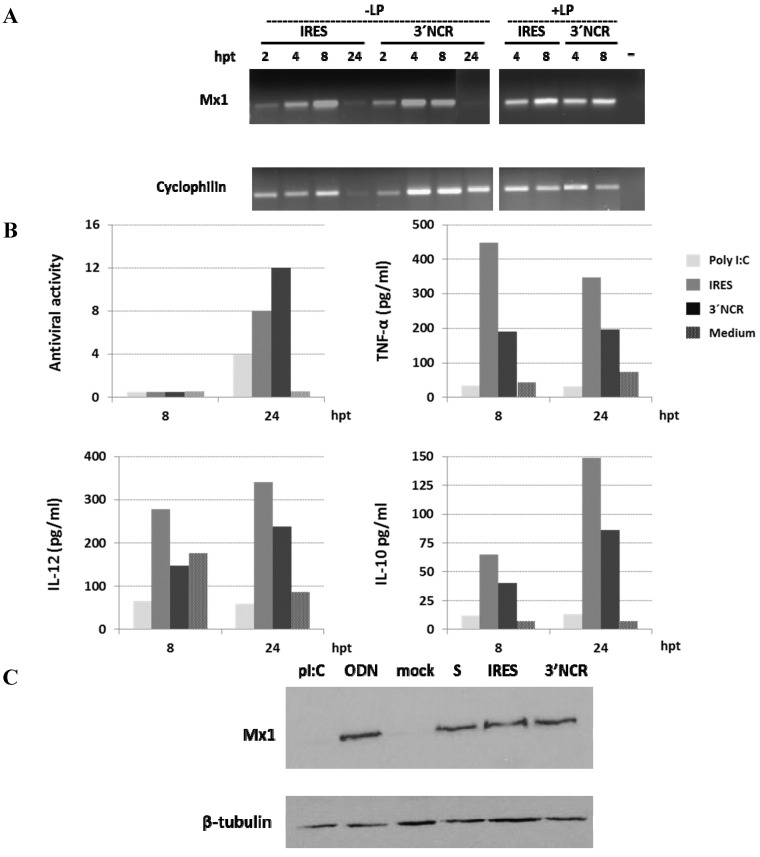
Analysis of the innate immune response of transfected PBMCs from a single pig (six month-old) over time. (**A**) Swine PBMCs were transfected with 20 μg/mL IRES or 3′NCR transcripts, with or without Lipofectin, and RNA was extracted at 2, 4, 8 or 24 h pt. Amplification of Mx1 and cyclophilin mRNAs by RT-PCR is shown; (**B**) Antiviral activity, TNFα, IL-12 and IL-10 levels in supernatants of swine PBMCs (2 × 10^6^) transfected with 3′NCR or IRES as in A or stimulated with poly I:C (10 μg/mL), and collected at 8 or 24 h pt. Mock transfections with culture medium and Lipofectin were performed as negative controls. Antiviral activity is expressed as the reciprocal of the highest dilution of supernatants from transfected PBMCs causing a 50% reduction of the cytophatic effect induced by infection with FMDV on IBRS-2 cells. The levels of the different cytokines were measured by ELISA; (**C**) Immunoblot detection of Mx1 in lysates of PBMCs transfected with S, IRES, 3′NCR transcripts (20 μg/mL) or mock-transfected using Lipofectin, or stimulated with poly I:C or ODN (both at 10 μg/mL). Cells were lysed 24 h pt. Tubulin was used for normalization.

To assess the potential effect of ncRNA inoculation in swine, we characterized the cytokine and antiviral responses induced in transfected swine PBMCs from a single animal up to 24 hpt. First, PBMCs were transfected with two different ncRNAs, IRES and 3′NCR transcripts. In both cases, induction of the Mx1 mRNA could be readily detected by RT-PCR, reaching the highest levels around 8 hpt (Figure 3A). This specific induction could also be observed even in the absence of Lipofectin. When supernatants from transfected PBMCs were tested for antiviral activity on IBRS-2 cells, significant levels of inhibition against FMDV infection were detected for both ncRNAs, particularly for the 3′NCR transcripts, at 24 hpt. (Figure 3B). Both RNAs induced higher levels of antiviral activity than the synthetic dsRNA analogue poly I:C. The specific induction of TNF-α, IL-10 and IL-12 was also determined by ELISA in the supernatants of PBMCs transfected with IRES or 3′NCR at 8 and 24 h pt. Higher levels of the three cytokines were detected in IRES-transfected PBMCs compared to those transfected with 3′NCR transcripts. No antiviral activity or cytokine induction could be detected in the absence of Lipofectin (not shown). To confirm the presence of Mx1 protein in lysates from transfected PBMCs, cells were transfected with all three ncRNAs (S, IRES and 3′NCR), or treated with poly I:C and class A CpG ODN 2216, which are TLR3/MDA5 and TLR9 specific ligands, respectively, as controls. After 24 h of transfection, Mx1 could be detected by immunoblot in ncRNA-transfected and ODN-treated PBMCs (Figure 3C).

**Figure 4:**
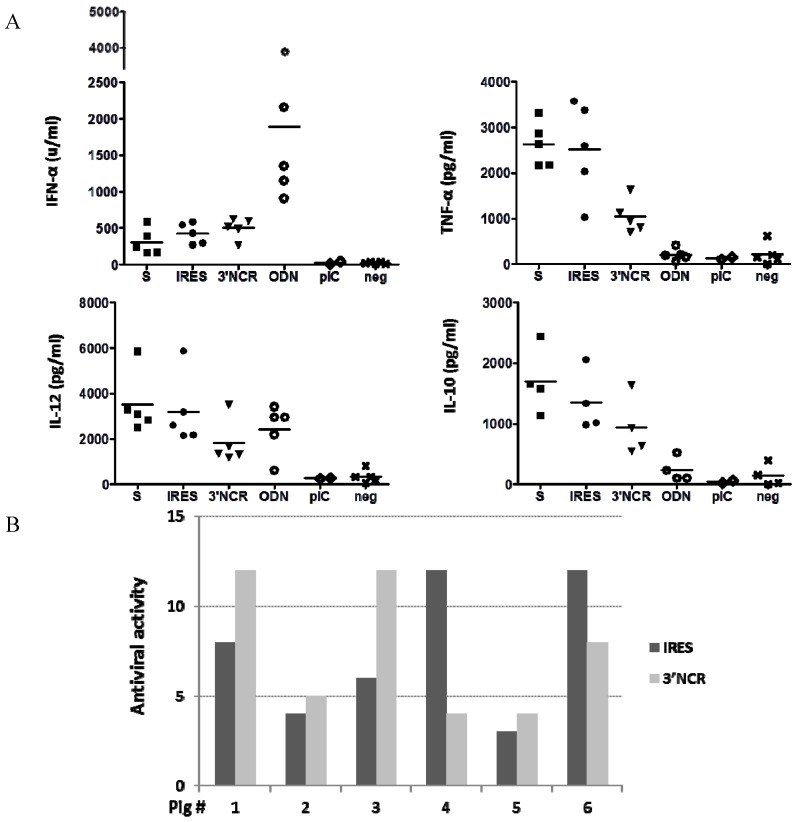
Innate responses in transfected PBMCs from different pigs and two independent experiments. (**A**) PBMCs were isolated from five 9 to 12-month-old pigs and transfected with 20 μg/mL S, IRES, 3′NCR, mock-transfected (negative control), or stimulated with ODN (10 μg/mL) or poly I:C (10 μg/mL) (*n* = 2). The levels of IFN-α, TNF-α, IL-12 and IL-10 were measured 24 h later by ELISA. Data correspond to individual animals for each group and analysis; (**B**) PBMCs were isolated from six three-month-old pigs and transfected with IRES, 3′NCR, or mock-transfected as above. Supernatants were tested for antiviral activity (expressed as in Figure 3) at 24 h pt. Individual pig numbers are indicated.

Since the above results were derived from PBMCs collected from a single animal, we extended the study to a larger number of animals. PBMCs were isolated individually from blood of five 9 to 12-month-old pigs and then transfected with S, IRES or 3′NCR RNAs. The levels of IFN-α, TNF-α, IL-10 and IL-12 in supernatants of the transfected PBMCs were analyzed by ELISA 24 h pt. Poly I:C and ODN 2216 were again used as controls. Although some inter-individual differences in response to ncRNA transfection could be observed, a consistent induction of IFN-α and cytokines was detected. No statistically significant differences in levels of IFN-α induction between the different ncRNAs were observed, though mean values for the 3′NCR were slightly higher (Figure 4A). Similarly, the analyzed cytokines were detected and the S and IRES transcripts seemed to be better inducers than the 3′NCR according to mean values, with statistically significant differences (*p* < 0.05) between TNF-α levels induced by S or IRES compared to those induced by transfection with the 3′NCR (Figure 4A). While treatment of PBMCs with the ODN 2216 resulted in higher levels of IFN-α induction compared to ncRNAs-transfected cells, remarkably higher levels of TNF-α and IL-10 were detected upon transfection with the ncRNAs relative to ODN-treated PBMCs; IL-12 was measured at similar levels in all cases with slight differences depending on the specific RNA. Poly I:C treatment of PBMCs of two animals did not result in induction of detectable levels of IFN-α or cytokines. In a different experiment, we tested the induction of antiviral activity in PBMCs isolated from six three-month-old pigs and transfected with IRES or 3′NCR transcripts. Again, although some inter-individual differences in response to transfection were observed, a consistent induction of antiviral activity was detected, resulting both RNAs equally effective, with some animals responding better to IRES and others to 3′NCR (Figure 4B). Taken together, these results reveal that the FMDV ncRNAs are able to elicit a robust innate immune response in porcine PBMCs.

### 3.4. Effect of ncRNA Dose and Enrichment in pDCs on Cytokine Production by Swine PBMCs

**Figure 5:**
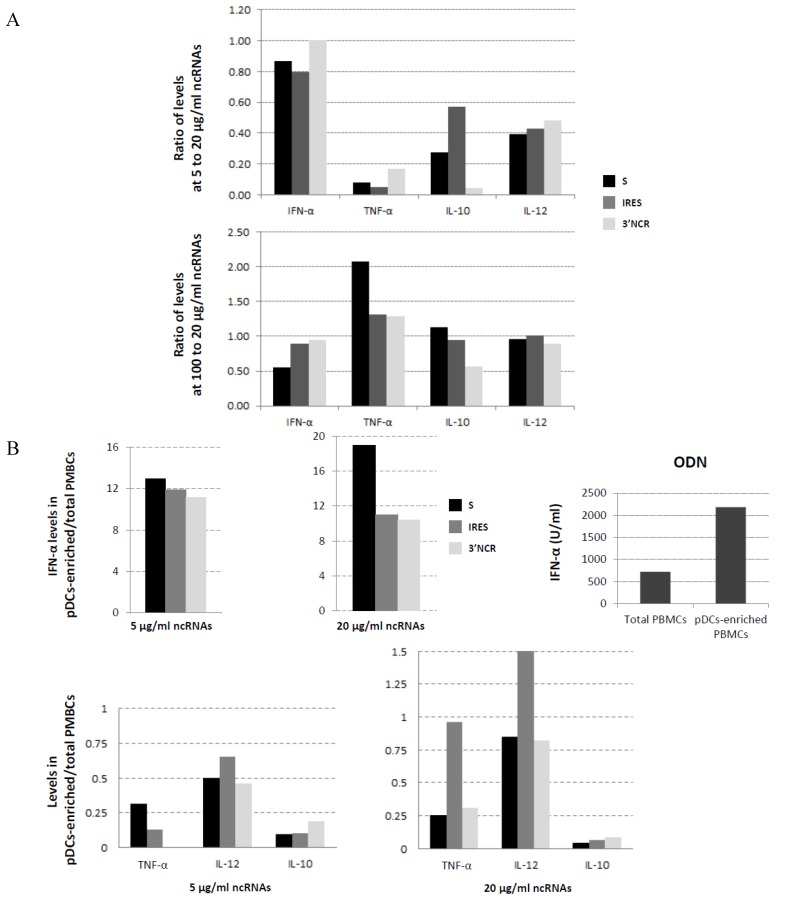
Effect of ncRNA dosage on cytokine induction in total swine PBMCs or after pDCs-enrichment. (**A**). PBMCs were transfected with S, IRES or 3′NCR at 5, 20 or 100 μg/mL and the IFN-α, TNF-α, IL10 and IL12 levels were measured by ELISA. The data shown are the ratios of the values obtained at 5 or 100 μg/mL for the indicated cytokine relative to those obtained at 20 μg/mL with the corresponding RNA; (**B**) Total PBMCs or PBMCs enriched in pDCs by negative selection were transfected with S, IRES or 3′NCR (at 5 or 20 μg/mL) or stimulated with 10 μg/mL ODN. Data indicate levels of IFN-α, TNF-α, IL-10 and IL-12 in pDCs-enriched PBMCs transfected with the ncRNAs relative to those in total PBMCs. IFN-α levels in total or pDCs-enriched PBMCs stimulated with ODN are also shown.

In all the ncRNAs transfection experiments above, an RNA concentration of 20 μg/mL had been used. To address the effect of higher, as well as lower RNA doses, on IFN-α and cytokines induction, we transfected PBMCs with S, IRES or 3′NCR ncRNAs at 5 or 100 μg/mL and calculated the ratios relative to those obtained with 20 μg/mL. As shown in Figure 5A, though IFN-α production was barely affected, lower levels of induction for the other cytokines were achieved using a four-fold lower RNA dose (5 μg/mL), and only a slight improvement was observed for TNF-α induction (two-fold) using a five-fold higher dose (100 μg/mL), particularly in S-transfected PBMCs. These results showed that the ncRNA-induced response was dose-dependent and confirmed 20 μg/mL of ncRNA as an appropriate transfection dose for the screening of cytokine induction levels.

Since pDCs are the main blood IFN-α producers in response to viral infections, playing a central role in antiviral immunity [31,32,33], we next assayed the response to ncRNA transfection of a PBMCs subset enriched in pDCs by negative selection, and compared it to that observed with total PBMCs. The negative selection procedure used yields around a 10-fold enrichment in pDCs corresponding to a 3%–6% of pDCs in the PBMC population. For that, two different concentrations of the S, IRES or 3′NCR RNAs were used for transfection and the induction profiles analyzed by ELISA, as above. Figure 5B shows the measured levels of IFN-α, TNF-α, IL-12 and IL-10 in pDC-enriched PBMCs relative to those in total PBMCs. At both RNA concentrations assayed, pDC-enriched PBMCs induced 10 to 19-fold higher levels of IFN-α upon transfection, with statistically highly significant differences (*p* < 0.0001) in all cases; in the case of the S transcript, those levels were 1.5 higher when the dose was increased from 5 to 20 μg/mL. In control cells treated with the ODN, three-fold higher levels of IFN-α were detected in pDC-enriched compared to total PBMCs. However, enrichment in pDCs abolished IL-10 induction dependent on ncRNA transfection and resulted detrimental for TNF-α except for the 20 μg/mL IRES. IL-12 induction was affected to a lesser extent as similar IL-12 levels were detected in pDC-enriched and total PBMCs transfected with 20 μg/mL of the three ncRNAs, and even a 1.5-fold increase was detected upon IRES transfection. Taken together, these results suggest that IFN-α production in ncRNA-transfected PBMCs is linked to pDCs.

### 3.5. The Innate Immune Response Induced by the FMDV ncRNAs in Porcine PBMCs is Significantly Decreased by Inhibition of Endosomal Acidification

**Figure 6:**
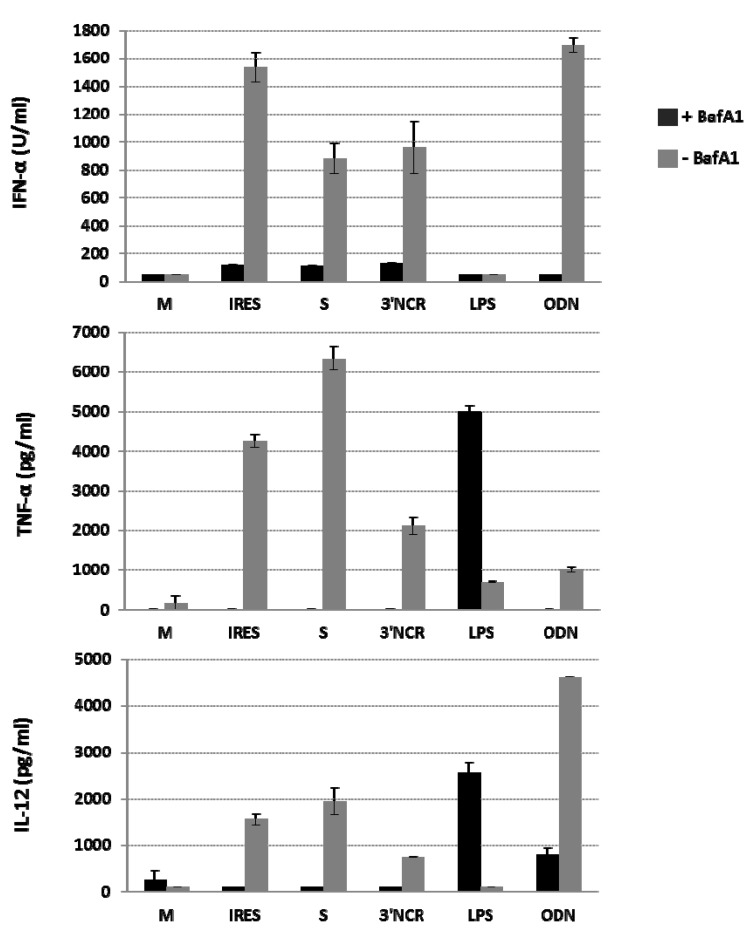
Effect of BafA1 treatment on cytokine induction in ncRNA-transfected PBMCs. Porcine PBMCs were treated with bafilomycin A1 (250 nM) 15 to 30 min prior to transfection or stimulation. IFN-α, TNF-α and IL-12 levels were measured by ELISA in total PBMCs 24 h after transfection with the ncRNAs (20 μg/mL) or stimulation with ODN (10 μg/mL) or LPS (0.5 μg/mL). Mock-transfections with medium were used as controls. Data are average values plus standard deviations from duplicates of two transfection experiments. Negative samples with OD readings ≤ the mean of blank wells were arbitrarily assigned values of 50, 25, and 100 for IFN-α, TNF-α, and IL-12, respectively, according to the detection limit for each cytokine.

IFN-α production in pDCs preferentially relies on the TLR pathway [34,35]. As responses induced by nucleic acid-sensing endosomal TLRs-3, -7/8, and -9 require intact endocytic pathways, to gain insight into the molecular mechanisms involved in FMDV ncRNAs sensing in swine PBMCs we tested the effect of bafilomycin A1, an inhibitor of endosomal acidification, on IFN-α, TNF-α and IL-12 induction as result of ncRNAs transfection. Control experiments were performed including the TLR-9 ligand ODN 2216, and bacterial lipopolysaccharide (LPS) as an agonist for TLR-4, localized to plasma membrane on the cell surface. The results are shown in Figure 6. As expected, bafilomycin abrogated IFN-α production in response to TLR-9 agonist ODN 2216. In the case of the ncRNAs, bafilomycin exerted a similar inhibitory effect on IFN-α induction though detectable levels could still be measured above the detection limit of the assay. No induction of IFN-α was detected in LPS-transfected PBMCs treated or not with the drug. Bafilomycin also abrogated TNF-α and IL-12 production in PBMCs stimulated with the ODN and ncRNA-transfected PBMCs. However, production of TNF-α and IL-12 in response to LPS was not inhibited by bafilomycin treatment. The sensitivity to bafilomycin A1 of the innate response triggered by the ncRNAs in PBMCs suggests their engagement to the endosomal TLRs pathway.

## 4. Discussion

Innate immune responses play a pivotal role in control and pathogenesis of viral infections [36]. Host recognition system of viral components may offer unique translational implications in medical approaches taking advantage of the innate immune function of PRRs to trigger cell autonomous responses. The concept of using targeted application of PAMPs to mimic a situation of viral infection is being extensively explored. In addition to its role in driving innate immune defenses, type-I IFNs play a major role in modulating the adaptive immune response [37,38,39,40]. Evidence is now emerging that many empiric vaccines and adjuvants inherently stimulate PRRs, highlighting the potential of vaccination strategies combining different PRRs ligands to stimulate polyvalent immune responses [41]. However, targeted immunomodulatory strategies will require knowledge of the virus-specific aspects of the pathway involved in sensing of the pathogen and subsequent induction of the immune response. In the case of FMDV, many efforts are being invested on improvement of currently used conventional inactivated vaccines, especially in terms of duration of the immunity, serotype-cross protection and shortening of the time required to induce protective immunity in vaccinated animals (around seven days) [42]. The FMDV ncRNAs are small synthetic non-infectious RNA transcripts corresponding in sequence to structural and functional domains in the 5′- and 3′-terminal non-coding regions of the viral genome. Their sequence and/or structure trigger a strong innate immune response inducing an antiviral activity in mice able to prevent viral spread and disease after inoculation with FMDV (ss(+)RNA picornavirus), West Nile virus (ss(+)RNA flavivirus) or Rift Valley fever virus (ss(−)RNA bunyavirus) [13,14,15]. In combination with an inactivated vaccine, the IRES transcripts proved to enhance the level and duration of specific antibodies promoting long-term protection after a single dose. Moreover, delivery of IRES ncRNA yield better results in terms of antibody levels, especially at early times post-vaccination, than a conventionally adjuvanted vaccine [20].

The encouraging results on the immunomodulatory effect of the ncRNAs led us to gain insight into the cellular pathways engaged to their sensing in host cells resulting in the innate response. As both RIG-I and MDA5 expression plasmids available corresponded to the human sequences, human kidney embryonic HEK 293 cells were used for the analysis. Unlike MDA5, expression of RIG-I led to a dose-dependent specific increase in the IFN-β promoter activity induced by transfection with each ncRNA, suggesting that the RLR pathway is involved by RIG-I signaling in sensing the ncRNAs in HEK 293 cells.

The paracrine propagation of the type-I IFN response triggered by transfection with the ncRNAs could be readily detected in SK6 as well as in swine PBMCs. Thus, Mx1 mRNA induction was observed by RT-PCR and the protein detected by immunoblot from 6 to 24 h pt. Porcine Mx1 overexpression has been shown to exert an inhibitory effect on FMDV and bovine viral diarrhea virus (BVDV) replication within 12 and 36 h post-infection [43], and protection against viral infection in cells of transgenic pigs expressing the Mx1 transgene has also been reported [44]. Mx1 has been shown to be upregulated in swine PBMCs of pigs infected with replication defective Ad5 expressing IFN-α at one and two days post-infection [45]. The broad spectrum of antiviral activities displayed by Mx1 makes it an interesting candidate gene to improve disease resistance in farm animals.

Since previous studies had been conducted in porcine cultured cells and mice, the response to the FMDV ncRNAs was analyzed in PBMCs from different pigs. Antiviral activity or IFN-α, as well as TNF-α, IL-10 and IL-12 production could be detected and measured in supernatants from transfected PBMCs in a dose-dependent manner and with different levels depending on the particular RNA and animal. TNF-α, a pro-inflammatory cytokine involved in recruitment of immune cells to the infection site, was detected at 8 h pt and then decreased at 24 h pt. IL-12 and IL-10 were detected at 8 h pt and their levels increased at 24 h pt. IL-12 plays a role in skewing the differentiation of CD4 helper T cells towards a Th1 response, producing IFN-Ɣ and other cytokines important in defense against intracellular pathogens, including viruses. IL-10, a cytokine with anti-inflammatory properties, has a central role in infection by limiting the immune response to pathogens and thereby preventing damage to the host reducing excessive immunopathology caused by inflammatory cytokines. Specific antiviral and/or cytokine responses were observed in PBMCs from all 12 pigs tested after transfection with the ncRNAs.This is in agreement with the previous reports describing the potent antiviral and immunostimulatory activities exerted *in vivo* by these RNA molecules. The IFN-α production observed was mainly linked to pDCs subset in the PBMCs population, known to play a central role in antiviral immunity [31,32,33].

Unlike cultured swine kidney SK6 cells, PBMCs are presumed to express several TLRs including nucleic acid sensing endosomal TLRs. Then, the putative sensing of the ncRNAs by TLRs was assessed by testing the effect on the induced innate response of treatment with bafilomycin A1 which inhibits endosomal acidification and, hence, signaling by endosomal TLRs—TLR3, 7, 8, and 9—prior to transfection. Using bafilomycin, we observed a strong inhibition of the innate response to the FMDV ncRNAs, with complete abrogation of TNF-α and IL-12 production. In contrast, no inhibitory effect was detected on cytokine production induced by LPS, a ligand of TLR4, which is located on the cell surface. Interestingly, though inhibitions above 90% on IFN-α production were observed in bafilomycin-treated cells in response to the ncRNAs, low levels of IFN-α could still be detected, unlike in those stimulated with the TLR-9 dependent ODN. These results suggest the involvement of the TLR pathway as a sensor system for the ncRNAs in swine PBMCs. Whether the residual IFN-α production in ncRNA-transfected PMBCs treated with the drug is dependent on RLR activation would require further research. Altogether, our findings provide evidence for the involvement of both TLR and cytosolic sensing of the FMDV ncRNAs and suggest that these molecules may have an immunostimulatory activity in livestock. These synthetic non-infectious molecules are able to trigger a broad innate immune response engaged to both RLR- and TLR-dependent pathways and have a potential application as immunomodulatory compounds in new antiviral and vaccine formulations. Those applications will require additional work on the activity of the particular RNA in the corresponding formulation context and delivery approach.
